# Proton pump inhibitor use and mortality in patients with cirrhosis: a meta-analysis of cohort studies

**DOI:** 10.1042/BSR20193890

**Published:** 2020-06-05

**Authors:** Xiaoli Wu, Daofu Zhang, Yuexiao Yu, Lianqing Lou, Xiaofei Li

**Affiliations:** 1 Department of Infectious Diseases, Yiwu Central Hospital, Yiwu 322000, China; 2 Department of Critical Care Medicine, Liaocheng Dongchangfu People’s Hospital, Liaocheng 252000, China

**Keywords:** Cirrhosis, Meta-analysis, Mortality, Proton pump inhibitor

## Abstract

**Background:** Proton pump inhibitor (PPI) is commonly used in patients with cirrhosis. However, some studies demonstrated that PPI use was associated with adverse outcome in patients with cirrhosis. We aimed to perform a meta-analysis of cohort studies to evaluate the association between PPI use and mortality in cirrhotic patients.

**Methods:** Relevant studies were obtained via search of PubMed and Embase databases. A randomized-effect model was used to pool the results. Subgroup analyses were performed to evaluate the source of heterogeneity.

**Results:** Overall, 21 cohort studies with 20,899 patients and 7457 death events were included. The pooled results with a randomized-effect model showed that PPI use was associated with significantly increased risk of mortality in patients with cirrhosis (adjusted relative risk [RR] = RR: 1.39, *P*<0.001) with considerable heterogeneity (*I*^2^=73%). Subgroup analyses showed that characteristics such as patient ethnicity, sample size, definition of PPI use, and complications of patients did not affect the association. However, the association between PPI use and mortality was independent of study characteristics including patient ethnicity, sample size, complications, definition of PPI use, and follow-up duration. However, the association between PPI use and mortality in cirrhotic patients was significant in retrospective studies (RR: 1.40, *P*<0.001), but not in prospective studies (RR: 1.34, *P*=0.33).

**Conclusions:** PPI use may be associated with moderately increased mortality in cirrhotic patients. Although prospective cohort studies are needed to validate our findings, PPI should only prescribed to cirrhotic patients with indications for the treatment.

## Introduction

Proton pump inhibitors (PPIs) are the first-line medications for the treatment of acid-related diseases, such as gastroesophageal reflux disease, peptic ulcer, and Zollinger-Ellison syndrome et al [1–3]. For patients with cirrhosis, PPIs are also commonly used. According to previous data, proportions of cirrhotic patients who were prescribed with PPIs varied from 30% to 80% [4]. However, the indications and clinical efficacy of PPIs in these patients remain to be validated, which highlights the possible overutilization of PPIs in patients with cirrhosis [5]. Subsequently, increasing evidence from epidemiological studies indicated that PPI use may be associated with adverse outcomes in cirrhotic patients, spontaneous bacterial peritonitis (SBP) [6–11], and hepatic encephalopathy (HE) [12,13]. However, it remains unknown whether PPI use affects mortality in these patients. Previous studies evaluating the association between PPI use and mortality risk in patients with cirrhosis showed inconsistent results [14–34]. An early meta-analysis published in 2015 showed that PPI use was not associated with increased mortality in patients with cirrhosis [11]. However, only four cohort studies [14,16,17,19] were included in the present study, and many related cohort studies have been published since the previous meta-analysis [18,20–34]. In addition, it remains unknown whether study design such as study design, ethnicity of the patients, definition of PPI use, or follow-up duration may affect the outcome. Therefore, we aimed to perform a meta-analysis to systematically evaluate the potential association between PPI use and mortality in patients with cirrhosis.

## Methods

The meta-analysis was performed in accordance with the MOOSE (Meta-analysis of Observational Studies in Epidemiology) [35] and Cochrane’s Handbook [36] guidelines.

### Literature search

Studies were identified via systematic search of electronic databases of PubMed and Embase via the following terms: (1) “proton pump inhibitor” OR “proton pump inhibitors” OR “acid suppressive therapy” OR “anti-secretory therapy” OR “PPI” OR “anti-ulcer agent” OR “antacid” OR “omeprazole” OR “esomeprazole” OR “lansoprazole” OR “pantoprazole” OR “rabeprazole” OR “ilaprazole” and (2) “cirrhosis” OR “cirrhotic” OR “liver fibrosis”. This extensive search strategy was used to avoid the potential missing of related studies. The search was limited to human studies in English. The reference lists of related original and review articles were also analyzed using a manual approach. The final literature search was performed on September 20, 2019.

### Study selection

The inclusion criteria for the studies were: (1) cohort studies published in full-length articles in English; (2) included patients with cirrhosis; (3) evaluated the association between PPI use and mortality risk in these patients; and (4) reported the relative risk for the association after adjustment of potential confounding factors. Reviews, editorials, preclinical studies, and studies irrelevant to the aim of current meta-analysis were excluded.

### Data extracting and quality evaluation

Literature search, data extraction, and quality assessment of the included studies were performed according to the predefined inclusion criteria by two independent authors (X.W. and X.L.). If discrepancies occurred, they were resolved by consensus of the two authors. The extracted data included: (1) name of first author, publication year and country where the study was performed; (2) study design characteristics; (3) ethnicity, characteristics, age, and gender of the participants; (4) definition of PPI use; (5) follow-up durations for cohort studies; and (6) variables that were adjusted when presenting the results. The quality of each study was evaluated using the Newcastle–Ottawa Scale [37] that ranges from 1 to 9 stars and judges each study regarding three aspects: selection of the study groups, the comparability of the groups, and the ascertainment of the outcome of interest.

### Statistical analyses

We used risk ratios (RRs) and their corresponding 95% confidence intervals (CIs) as the general measure for the association between PPI use and mortality in patients with cirrhosis. Data of RRs and their corresponding stand errors (SEs) were calculated from 95% CIs or *P* values, and were logarithmically transformed to stabilize variance and normalized the distribution [36]. The Cochrane’s *Q* test and *I*^2^ test were used to evaluate the heterogeneity among the include cohort studies [38]. A significant heterogeneity was considered if *I*^2^>50%. We used a randomized-effect model to synthesize the RR data because this model is considered as a more generalized method that incorporates of the potential heterogeneity [36]. Sensitivity analyses, by removing individual study one at a time, were performed to test the robustness of the results [39]. Predefined subgroup analyses were performed to evaluate the influences of study characteristics on the outcome, including study design, patient ethnicity, patient characteristics, sample size, definition of PPI use, and follow-up durations. The potential publication bias was assessed by funnel plots with the Egger regression asymmetry test [40]. We used the RevMan (Version 5.1; Cochrane Collaboration, Oxford, U.K.). and STATA software for the meta-analysis and statistics.

## Results

### Literature search

**Figure 1:**
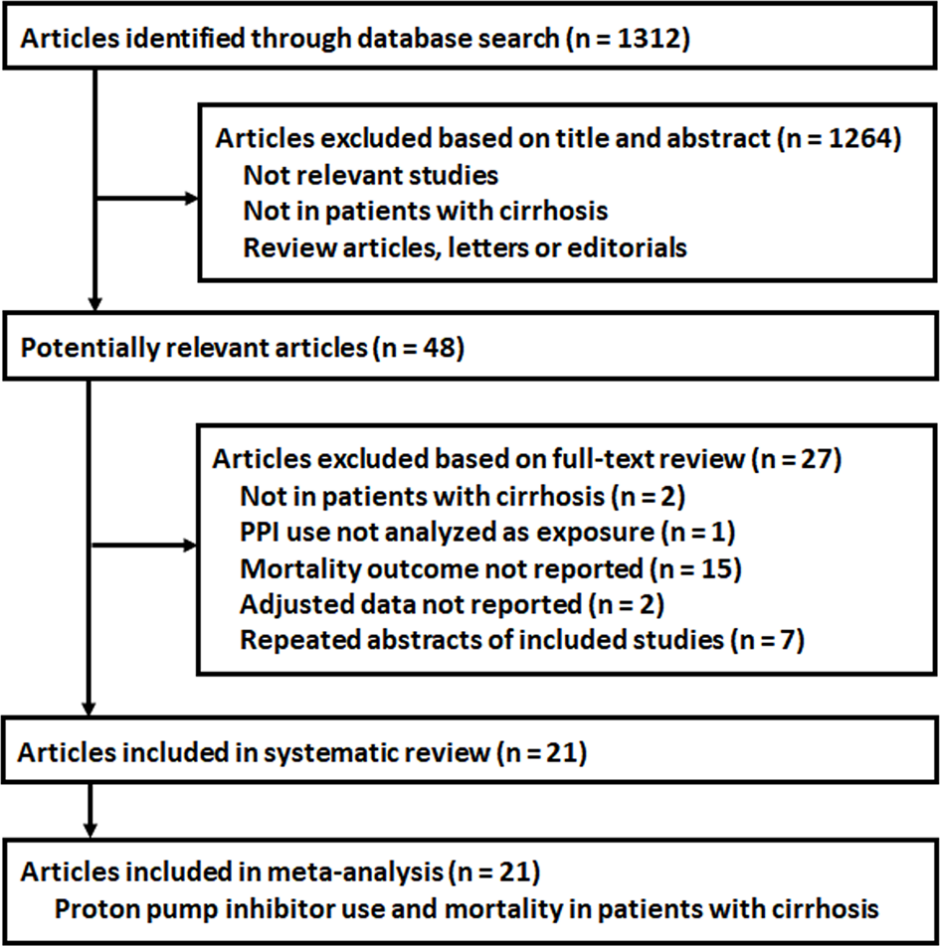
Flowchart of database search and study identification

The process of database search is summarized in Figure 1. Briefly, 1312 articles were found via initial literature search of the PubMed and Embase databases, and 1264 were excluded through screening of the titles and abstracts mainly because they were not relevant to the purpose of the meta-analysis. Subsequently, 48 potential relevant records underwent full-text review. Of these, 27 were further excluded because two of them did not include patients with cirrhosis, one did not analyze PPI in cirrhotic patients, 15 did not report mortality outcome, two did not provide adjusted data for the association, and the other seven were abstracts of the already included studies. Finally, 21 cohort studies were included [14–34].

### Study characteristics and quality evaluation

The characteristics of the included studies are summarized in Table 1. Overall, 21 cohort studies with 20,899 patients and 7457 death events were included, of which 16 were retrospective cohort studies [16–19,22–33], while the other five were prospective cohort [14,15,20,21,34]. As for the ethnicity of the patients, 12 studies included Caucasian patients [15,16,18,20–22,24,28,29,32–34], 7 included Asians [17,19,23,25,26,30,31], and the remaining 2 included patients with mixed ethnicity [14,27]. Most of the studies included patients with hospitalized patients with cirrhosis without serious clinical complications [14–18,20–24,28–30,32,34], while six studies included cirrhotic patients with SBP [19,26], HE [25], *Clostridium difficile* infection [27], pneumonia [31], and after transjugular intrahepatic portosystemic shunt [33], respectively. The mean ages of the patients varied from 53 to 63 years, and the proportions of male patients varied from 53% to 77%. PPI use was defined as concurrent use at the admission in 13 studies [14,16–18,20,22,25–29,31,34], and any use during follow up in eight studies [15,19,21,23,24,30,32,33]. The follow-up durations varied from within hospitalization to 60 months. Variables including age, gender, comorbidities, and scale for disease severity, such as the Child-Pugh class or the Model for End-stage liver Disease (MELD) score were adjusted when presenting the association between PPI use and mortality. The qualities of the included cohorts were generally good, with NOS scores ranging between 7 and 9 (Table 2).

**Table 1:** Characteristics of the included studies

Study	Country	Design	Ethnicity	Patient characteristics	Sample size	Mean age	Male	Definitions of PPI use	Follow-up durations	Death (*n*)	Variables adjusted	NOS
						Years	%		Months			
van Vlerken 2012	The Netherlands	PC	Caucasian	Cirrhotic patients with ascites	84	55.0	67.0	Any PPI use within follow-up	28	17	Age, gender, and Child-Pugh class	7
Bajaj 2012	US	PC	Mixed	Cirrhotic patients	207	55.0	59.9	Current PPI use at admission	1	49	Age, gender, MELD score, albumin, serum sodium, SOFA score	8
de Vos 2013	Belgium	RC	Caucasian	Cirrhotic patients with ascites	51	57.5	68.6	Current use for at least 2 weeks by medical record review	12	40	Age, gender, INR, MELD score, and Child-Pugh class	7
Min 2014	Korea	RC	Asian	Cirrhotic patients with SBP	134	58	73.9	Any PPI use during hospitalization	Within hospitalization	41	Age, gender, serum sodium, MELD score, and Child-Pugh class	8
Kwon 2014	Korea	RC	Asian	Cirrhotic patients with ascites	533	62.3	75.4	Current use of PPI within 30 days before admission	1	175	Age, gender, and MELD score	8
Mandorfer 2014	Austria	RC	Caucasian	Cirrhotic patients with ascites	607	57.5	70.0	Current use on admission by medical record review	10	358	Age, gender, and MELD score	8
Dultz 2015	Germany	PC	Caucasian	Cirrhotic patients	272	57.0	66.9	Current PPI use at admission	9	86	Age, gender, etiology of cirrhosis, and MELD Score	8
Merli 2015	Italy	PC	Caucasian	Cirrhotic patients	400	61.5	70.3	Any PPI use during hospitalization	Within hospitalization	39	Age, gender, and MELD score	7
Cole 2016	U.K.	RC	Caucasian	Cirrhotic patients	198	56.0	65.2	Current PPI use at discharge	23	38	Age, gender, and MELD score	7
Kim 2017	Korea	RC	Asian	Cirrhotic patients with a previous SBP	307	57.6	77.1	Any PPI use for at least 1 week during follow-up	Within hospitalization	91	Age, gender, etiology of liver disease, serum sodium, SCr, and Child-Pugh class	7
Miozzo 2017	Brazil	RC	Caucasian	Cirrhotic patients	258	53.6	58.2	Any PPI use during follow-up	30	155	Age, gender, and MELD score	7
Hung 2018a	China	RC	Asian	Cirrhotic patients with SBP	2574	60.9	71.8	Current PPI use during hospitalization	12	1785	Age, gender, and MELD score	7
Hung 2018b	China	RC	Asian	Cirrhotic patients with HE	5020	62.5	68.0	Current PPI use during hospitalization	12	3210	Age, gender, etiology of cirrhosis, and MELD score	7
Smith 2018	US	RC	Mixed	Cirrhotic patients with CDI	400	58.5	60.0	Current PPI use at admission	30	44	Age, gender, serum albumin, and MELD score	7
Tergast 2018	Germany	RC	Caucasian	Cirrhotic patients with ascites	613	56.1	62.0	Current PPI use within 7 days before admission	1	121	Age, gender, and MELD score	8
Janka 2019	Hungary	RC	Caucasian	Cirrhotic patients	350	56.0	53.7	Regular use of PPI for at least 80% of follow-up periods based on medical chart review	60	147	Age, gender, and MELD score	7
Nardelli 2019	Italy	PC	Caucasian	Cirrhotic patients	310	62.2	71.3	Current PPI use at least 4 weeks before admission	14	112	Age, gender, serum albumin, serum sodium, and MELD score	9
Lewis 2019	US	RC	Caucasian	Cirrhotic patients after TIPS	2284	56.4	66. 1	Any PPI use at follow-up	13	115	Age, gender, and MELD score	8
Dam 2019	Denmark	RC	Caucasian	Cirrhotic patients	1198	57.5	70.0	Current PPI use at admission	3	81	Age, gender, MELD score, history of variceal bleeding, and history of HE	7
Hung 2019	China	RC	Asian	Cirrhotic patients with pneumonia	4804	63.0	71.2	Current PPI use during hospitalization	3	638	Age, gender, etiology of cirrhosis, and MELD score	7
De Roza 2019	Singapore	RC	Asian	Cirrhotic patients	295	62.8	68.1	Any PPI use at follow-up	6	115	Age, gender, etiology of cirrhosis, history of variceal bleeding and HE, and MELD score	8

Abbreviations: CDI, *Clostridium difficile* Infection; DM, diabetes mellitus; HE, hepatic encephalopathy; INR, international normalized ratio; MELD, Model for End-stage liver Disease; NOS, the Newcastle–Ottawa Score; PC, prospective cohort; PPI, proton pump inhibitor; RC, retrospective cohort; SCr, serum creatinine; SBP, spontaneous bacterial peritonitis; TIPS, transjugular intrahepatic portosystemic shunt.

**Table 2:** Details of study quality evaluation by the Newcastle–Ottawa Scale

Study	Representativeness of the exposed cohort	Selection of the non-exposed cohort	Ascertainment of exposure	Outcome not present at baseline	Control for age and gender	Control for other confounding factors	Assessment of outcome	Enough long follow-up duration	Adequacy of follow-up of cohorts	Total
van Vlerken 2012	**0**	**1**	**0**	**1**	**1**	**1**	**1**	**1**	**1**	**7**
Bajaj 2012	**1**	**1**	**0**	**1**	**1**	**1**	**1**	**1**	**1**	**8**
de Vos 2013	**0**	**1**	**1**	**1**	**1**	**1**	**0**	**1**	**1**	**7**
Min 2014	**0**	**1**	**1**	**1**	**1**	**1**	**1**	**0**	**1**	**7**
Kwon 2014	**1**	**1**	**0**	**1**	**1**	**1**	**1**	**1**	**1**	**8**
Mandorfer 2014	**1**	**1**	**1**	**1**	**1**	**1**	**1**	**1**	**0**	**8**
Dultz 2015	**1**	**1**	**0**	**1**	**1**	**1**	**1**	**1**	**1**	**8**
Merli 2015	**1**	**1**	**1**	**1**	**1**	**1**	**0**	**0**	**1**	**7**
Cole 2016	**0**	**1**	**0**	**1**	**1**	**1**	**1**	**1**	**1**	**7**
Kim 2017	**1**	**0**	**0**	**1**	**1**	**1**	**1**	**1**	**1**	**7**
Miozzo 2017	**0**	**1**	**0**	**1**	**1**	**1**	**1**	**1**	**1**	**7**
Hung 2018a	**1**	**0**	**0**	**1**	**1**	**1**	**1**	**1**	**1**	**7**
Hung 2018b	**1**	**0**	**0**	**1**	**1**	**1**	**1**	**1**	**1**	**7**
Smith 2018	**0**	**1**	**0**	**1**	**1**	**1**	**1**	**1**	**1**	**7**
Tergast 2018	**1**	**1**	**1**	**1**	**1**	**1**	**0**	**1**	**1**	**8**
Janka 2019	**0**	**1**	**1**	**0**	**1**	**1**	**1**	**1**	**1**	**7**
Nardelli 2019	**1**	**1**	**1**	**1**	**1**	**1**	**1**	**1**	**1**	**9**
Lewis 2019	**0**	**1**	**1**	**1**	**1**	**1**	**1**	**1**	**1**	**8**
Dam 2019	**1**	**1**	**0**	**1**	**1**	**1**	**1**	**0**	**1**	**7**
Hung 2019	**1**	**0**	**0**	**1**	**1**	**1**	**1**	**1**	**1**	**7**
De Roza 2019	**1**	**1**	**1**	**1**	**1**	**1**	**1**	**0**	**1**	**8**

### Association between PPI use and mortality in patients with cirrhosis

**Figure 2:**
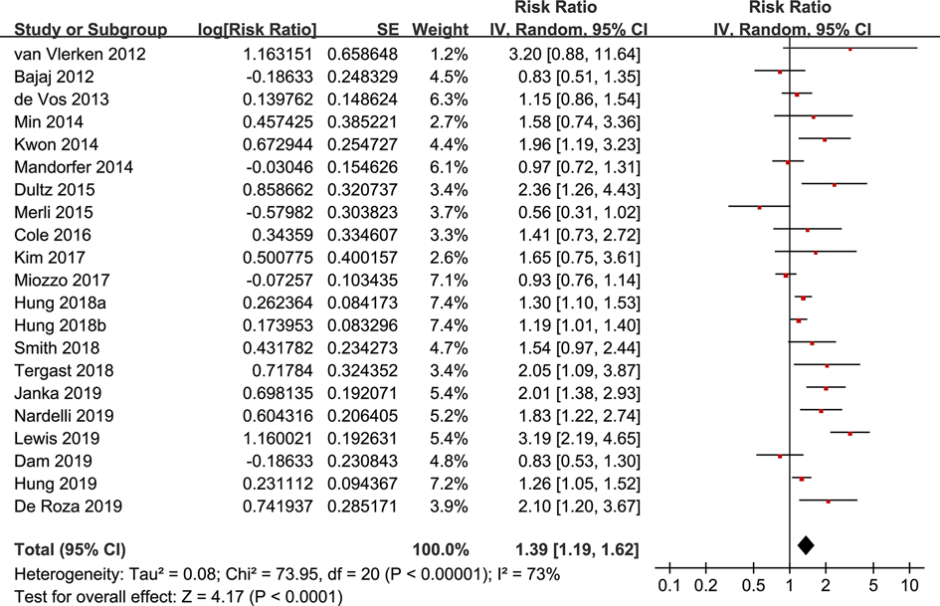
Forest plots for the meta-analysis of the association between PPI use and mortality in cirrhotic patients

Meta-analysis with a randomized-effect model including 21 studies showed that use of PPI was significantly associated with mortality in cirrhotic patients (RR: 1.39, 95% CI: 1.19–1.62, *P*<0.001; Figure 2) with significant heterogeneity (*P* for Cochrane’s *Q* test < 0.001, *I*^2^=73%). Results of sensitivity analyses by omitting one dataset at a time did not significantly change the results (RR: 1.31–1.43, *P* all < 0.001; Table 3). Subgroup analyses according to the study design showed that the association between PPI use and mortality risk in patients with cirrhosis was significant in retrospective cohort studies (16 studies, RR: 1.40, 95% CI: 1.20–1.64, *P*<0.001), but not in prospective cohort studies (five studies, RR: 1.34, 95% CI: 0.75–2.40, *P*=0.33; Table 4). However, the difference between the subgroups was not significant (*P*=0.88). The association between PPI use and mortality in cirrhotic patients was significant and not affected by study characteristics including patient ethnicity, with or without severe complications, sample size, definition of PPI use, or follow-up durations (Table 4).

**Table 3:** Results of sensitivity analysis

Studies omitted	RR	95% CI	*I*^2^	*P* for effect
van Vlerken 2012	1.37	1.18–1.60	74%	<0.001
Bajaj 2012	1.42	1.21–1.66	73%	<0.001
de Vos 2013	1.41	1.20–1.66	74%	<0.001
Min 2014	1.38	1.18–1.62	74%	<0.001
Kwon 2014	1.37	1.17–1.60	73%	<0.001
Mandorfer 2014	1.42	1.21–1.67	73%	<0.001
Dultz 2015	1.36	1.17–1.59	73%	<0.001
Merli 2015	1.43	1.23–1.67	71%	<0.001
Cole 2016	1.39	1.19–1.63	74%	<0.001
Kim 2017	1.38	1.18–1.62	74%	<0.001
Miozzo 2017	1.43	1.22–1.67	70%	<0.001
Hung 2018a	1.40	1.18–1.67	74%	<0.001
Hung 2018b	1.41	1.19–1.68	74%	<0.001
Smith 2018	1.38	1.18–1.62	74%	<0.001
Tergast 2018	1.37	1.17–1.60	74%	<0.001
Janka 2019	1.36	1.16–1.59	72%	<0.001
Nardelli 2019	1.37	1.17–1.60	73%	<0.001
Lewis 2019	1.31	1.14–1.50	62%	<0.001
Dam 2019	1.42	1.22–1.67	73%	<0.001
Hung 2019	1.40	1.19–1.67	74%	<0.001
De Roza 2019	1.36	1.17–1.59	73%	<0.001

Abbreviations: CI, confidence interval; RR, risk ratio.

**Table 4:** Subgroup analyses for the meta-analysis of the association between PPI use and mortality in patients with cirrhosis

Characteristics	Dataset number	RR (95% CI)	*P* for subgroup effect	*I*^2^	*P* for subgroup difference
Study design					
PC	5	1.34 [0.75, 2.40]	0.33	79%	
RC	16	1.40 [1.20, 1.64]	<0.001	73%	0.88
Ethnicity					
Caucasian	12	1.42 [1.07, 1.90]	0.02	83%	
Asian	7	1.32 [1.18, 1.48]	<0.001	17%	0.63
Patient characteristics					
With complications or after procedure	6	1.50 [1.19, 1.89]	0.001	79%	
Without complications	15	1.34 [1.08, 1.67]	0.008	71%	0.49
Number of participants					
>500	11	1.33 [1.03, 1.72]	0.03	71%	
≤500	10	1.45 [1.18, 1.78]	0.004	76%	0.61
Definition of PPI use					
Current PPI use at admission	13	1.28 [1.13, 1.46]	<0.001	48%	
Any PPI use during follow-up	7	1.60 [1.02, 2.53]	0.04	86%	0.35
Follow-up durations					
≤1 month	9	1.19 [1.01, 1.40]	0.04	70%	
>1 month	15	1.42 [1.20, 1.68]	<0.001	76%	0.15

Abbreviations: CI, confidence interval; PPI, proton pump inhibitor; PR, prospective cohort; RC, retrospective cohort; RR, risk ratio.

### Publication bias

**Figure 3:**
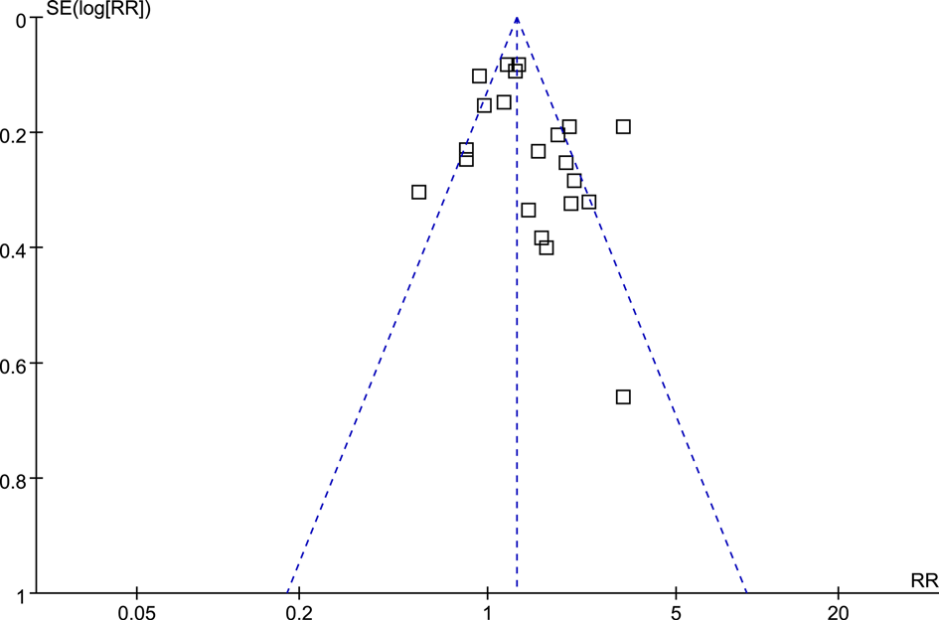
Funnel plots for the meta-analysis of the association between PPI use and mortality in cirrhotic patients

The funnel plots regarding the association between PPI use and mortality in cirrhotic patients were shown in Figure 3. The funnel plots were symmetry on visual inspection, suggesting low chance of significant publication bias. Results of Egger’s regression test also suggested that no significant publication bias (*P*=0.65).

## Discussion

Results of this meta-analysis showed that PPI use was associated with moderately increased mortality in patients with cirrhosis. The association between PPI use and mortality in cirrhotic patients seemed to be independent of demographic factors and scales indicating the disease severity because we included data after adjustment of these confounding factors. Moreover, sensitivity analyses by omitting one study at a time indicated the robustness of the outcome. Subgroup analyses also suggested that the association between PPI use and mortality in cirrhotic patients was consistent in different subgroups according to study characteristics including patient ethnicity, sample size, with or without patient complications, definition of PPI use, and follow-up durations. Although the association between PPI use and mortality was significant in subgroup of retrospective studies but not in subgroups of prospective studies, the difference between the subgroups was not significant. Taken together, these results indicated that PPI use may be associated with increased mortality risk in patients with cirrhosis. Although these findings should be validated in large-scale prospective studies, considering the potential overutilization of PPI in cirrhotic patients, carefully selection of cirrhotic patients with appropriate indications for PPI therapy is recommended before using the medication.

An early meta-analysis published in 2016 failed to show a significant association between PPI use and mortality risk in cirrhotic patients [11]. As mentioned by the authors, the limited number of available studies and sample size may lead to statistical inadequacy of the meta-analysis to reach a positive outcome. Our meta-analysis, by including 21 available cohort studies with 20,899 patients, showed that PPI use was associated with increased mortality in cirrhotic patients. Despite of the large number of studies included, our study has the following strengths. First, we only included article published in peer-reviewed journal, which therefore is of low risk of selection bias. Second, RRs data after adjustment of multiple confounding factors were included and combined, which therefore may provide an independent association between PPI use and mortality risk in cirrhosis patients. Third, we performed exhausting sensitivity and subgroup analyses to evaluate the potential influences of individual study and study characteristics on the outcome. These studies confirmed the robustness of the meta-analysis result, which was not likely to be driven by a single study or affected by study characteristics including patient ethnicity, sample size, with or without complications, definition of PPI, and follow-up durations. Although we found that the association between PPI use and mortality risk was significant in retrospective studies but not in prospective studies, difference between the subgroups was not statistically. Moreover, only five prospective cohorts were available. Therefore, it was likely that the non-significant association between PPI use and mortality risk in subgroup of prospective cohorts may probably be attributed to limited studies available. Since retrospective cohort studies is exposed to recall bias [41], large-scale prospective cohort studies are needed to validate our findings. Collectively, results of this meta-analysis support that PPI is associated with increased mortality in cirrhotic patients. Considering the potential overutilization of PPIs in patients with cirrhosis [5], as well as the potential safety consideration related to the long-term use of PPIs [3], decision for the prescription of PPIs in cirrhotic patients should only be made after carefully evaluating the appropriate indications and balancing the benefits and potential risk of adverse outcomes in these patients.

The reasons and mechanisms for the increased mortality risk associated with PPI use in cirrhotic patients may be multifactorial. First, PPIs, by suppressing gastric acids, may cause bacterial overgrowth in both stomach and intestine [42,43]. Subsequently, the impaired intestinal epithelial barrier in patients with cirrhosis [44] may facilitate the translocation of the overgrown bacteria to mesenteric lymph nodes, which finally leads to SBP and other infections [45]. Moreover, PPIs may also contribute to the gut dysbiosis that generally exists in patients with cirrhosis, whereas altered gut microbiota can induce or exacerbate HE [46,47], another complication that has been related to increased mortality risk in cirrhotic patients [12]. In addition, studies in overall population showed that PPI use may be associated with increased risks of cardiovascular adverse events [48], stroke [49], and fractures [50], which may also lead to increased mortality. Studies regarding the safety of PPI use in various populations, particularly the long-term use of PPI, should be performed to confirm these mechanisms.

Our study has limitations which should be considered when interpreting the results. First, significant heterogeneity exists among the included studies. Although exploring subgroup analyses were performed to evaluate the potential source of heterogeneity, the results of subgroup analyses should be interpreted with caution since the numbers of available studies were limited for each stratum of subgroups. Moreover, since the individual patient data were not available, we could only perform subgroup analyses based on study-level data. The influences of patient and study characteristics on the association between PPI use and mortality in cirrhotic patients should be further analyzed in future studies. Second, although we included studies with adjusted data for the association between PPI use and mortality, we could not exclude the existence of residual factors which may confound the association. Third, a causative association between PPI use and mortality in cirrhotic patients should not be derived based on our finding since the present study was a meta-analysis of observational studies. Randomized controlled trials should be considered to validate the influence of PPI on clinical outcomes in cirrhosis patients. Finally, we could not determine whether the dosages, durations, or individual PPI medications may affect the clinical outcomes of cirrhotic patients differently. Future studies are also warranted in this regard.

## Conclusions

In conclusion, results of our meta-analysis indicated that PPI use may be associated with increased mortality risk in cirrhotic patients. Although these findings should be validated in large-scale prospective studies, considering the potential overutilization of PPI in cirrhotic patients, carefully selection of cirrhotic patients with appropriate indications for PPI therapy is recommended before using the medication.

## Abbreviations

CDI: *Clostridium difficile* Infection
DM: diabetes mellitus
HE: hepatic encephalopathy
INR: international normalized ratio
MELD: Model for End-stage liver Disease
NOS: the Newcastle–Ottawa Score
PC: prospective cohort
PPI: proton pump inhibitor
RC: retrospective cohort
SBP: spontaneous bacterial peritonitis
SCr: serum creatinine
TIPS: transjugular intrahepatic portosystemic shunt

## References

[1] Vaezi M.F., Yang Y.X. and Howden C.W. (2017) Complications of Proton Pump Inhibitor Therapy. Gastroenterology 153, 35–48 10.1053/j.gastro.2017.04.047 28528705

[2] Weersink R.A., Bouma M., Burger D.M. et al. (2018) Safe use of proton pump inhibitors in patients with cirrhosis. Br. J. Clin. Pharmacol. 84, 1806–1820 10.1111/bcp.13615 29688583 PMC6046475

[3] Corley D.A. (2019) Safety and Complications of Long-Term Proton Pump Inhibitor Therapy: Getting Closer to the Truth. Gastroenterology 157, 604–607 10.1053/j.gastro.2019.07.039 31378636

[4] Chavez-Tapia N.C., Tellez-Avila F.I., Garcia-Leiva J. and Valdovinos M.A. (2008) Use and overuse of proton pump inhibitors in cirrhotic patients. Med. Sci. Monit. 14, CR468–CR472 18758417

[5] Heidelbaugh J.J., Kim A.H., Chang R. and Walker P.C. (2012) Overutilization of proton-pump inhibitors: what the clinician needs to know. Therap. Adv. Gastroenterol. 5, 219–232 10.1177/1756283X12437358 22778788 PMC3388523

[6] Trikudanathan G., Israel J., Cappa J. and O'Sullivan D.M. (2011) Association between proton pump inhibitors and spontaneous bacterial peritonitis in cirrhotic patients - a systematic review and meta-analysis. Int. J. Clin. Pract. 65, 674–678 10.1111/j.1742-1241.2011.02650.x 21564440

[7] Siple J.F., Morey J.M., Gutman T.E., Weinberg K.L. and Collins P.D. (2012) Proton pump inhibitor use and association with spontaneous bacterial peritonitis in patients with cirrhosis and ascites. Ann. Pharmacother. 46, 1413–1418 10.1345/aph.1R174 23032651

[8] Deshpande A., Pasupuleti V., Thota P. et al. (2013) Acid-suppressive therapy is associated with spontaneous bacterial peritonitis in cirrhotic patients: a meta-analysis. J. Gastroenterol. Hepatol. 28, 235–242 10.1111/jgh.12065 23190338

[9] Khan M.A., Kamal S., Khan S., Lee W.M. and Howden C.W. (2015) Systematic review and meta-analysis of the possible association between pharmacological gastric acid suppression and spontaneous bacterial peritonitis. Eur. J. Gastroenterol. Hepatol. 27, 1327–1336 10.1097/MEG.0000000000000448 26313401

[10] Xu H.B., Wang H.D., Li C.H. et al. (2015) Proton pump inhibitor use and risk of spontaneous bacterial peritonitis in cirrhotic patients: a systematic review and meta-analysis. Genet. Mol. Res. 14, 7490–7501 10.4238/2015.July.3.25 26214428

[11] Yu T., Tang Y., Jiang L., Zheng Y., Xiong W. and Lin L. (2016) Proton pump inhibitor therapy and its association with spontaneous bacterial peritonitis incidence and mortality: A meta-analysis. Dig. Liver Dis. 48, 353–359 10.1016/j.dld.2015.12.009 26795544

[12] Ma Y.J., Cao Z.X., Li Y. and Feng S.Y. (2019) Proton pump inhibitor use increases hepatic encephalopathy risk: A systematic review and meta-analysis. World J. Gastroenterol. 25, 2675–2682 10.3748/wjg.v25.i21.2675 31210718 PMC6558435

[13] Tantai X.X., Yang L.B., Wei Z.C. et al. (2019) Association of proton pump inhibitors with risk of hepatic encephalopathy in advanced liver disease: A meta-analysis. World J. Gastroenterol. 25, 2683–2698 10.3748/wjg.v25.i21.2683 31210719 PMC6558434

[14] Bajaj J.S., O'Leary J.G., Reddy K.R. et al. (2012) Second infections independently increase mortality in hospitalized patients with cirrhosis: the North American consortium for the study of end-stage liver disease (NACSELD) experience. Hepatology 56, 2328–2335 10.1002/hep.25947 22806618 PMC3492528

[15] van Vlerken L.G., Huisman E.J., van Hoek B. et al. (2012) Bacterial infections in cirrhosis: role of proton pump inhibitors and intestinal permeability. Eur. J. Clin. Invest. 42, 760–767 10.1111/j.1365-2362.2011.02643.x 22288900

[16] de Vos M., De Vroey B., Garcia B.G. et al. (2013) Role of proton pump inhibitors in the occurrence and the prognosis of spontaneous bacterial peritonitis in cirrhotic patients with ascites. Liver Int. 33, 1316–1323 10.1111/liv.12210 23730823

[17] Kwon J.H., Koh S.J., Kim W. et al. (2014) Mortality associated with proton pump inhibitors in cirrhotic patients with spontaneous bacterial peritonitis. J. Gastroenterol. Hepatol. 29, 775–781 10.1111/jgh.12426 24219827

[18] Mandorfer M., Bota S., Schwabl P. et al. (2014) Proton pump inhibitor intake neither predisposes to spontaneous bacterial peritonitis or other infections nor increases mortality in patients with cirrhosis and ascites. PLoS ONE 9, e110503 10.1371/journal.pone.0110503 25369194 PMC4219684

[19] Min Y.W., Lim K.S., Min B.H. et al. (2014) Proton pump inhibitor use significantly increases the risk of spontaneous bacterial peritonitis in 1965 patients with cirrhosis and ascites: a propensity score matched cohort study. Aliment. Pharmacol. Ther. 40, 695–704 10.1111/apt.12875 25078671

[20] Dultz G., Piiper A., Zeuzem S., Kronenberger B. and Waidmann O. (2015) Proton pump inhibitor treatment is associated with the severity of liver disease and increased mortality in patients with cirrhosis. Aliment. Pharmacol. Ther. 41, 459–466 10.1111/apt.13061 25523381

[21] Merli M., Lucidi C., Di Gregorio V. et al. (2015) The chronic use of beta-blockers and proton pump inhibitors may affect the rate of bacterial infections in cirrhosis. Liver Int. 35, 362–369 10.1111/liv.12593 24836902

[22] Cole H.L., Pennycook S. and Hayes P.C. (2016) The impact of proton pump inhibitor therapy on patients with liver disease. Aliment. Pharmacol. Ther. 44, 1213–1223 10.1111/apt.13827 27774677

[23] Kim J.H., Lim K.S., Min Y.W. et al. (2017) Proton pump inhibitors do not increase the risk for recurrent spontaneous bacterial peritonitis in patients with cirrhosis. J. Gastroenterol. Hepatol. 32, 1064–1070 10.1111/jgh.13637 28449345

[24] Miozzo S.A.S., John J.A., Appel-da-Silva M.C., Dossin I.A., Tovo C.V. and Mattos A.A. (2017) Influence of proton pump inhibitors in the development of spontaneous bacterial peritonitis. World J. Hepatol. 9, 1278–1285 10.4254/wjh.v9.i35.1278 29290909 PMC5740091

[25] Hung T.H., Lee H.F., Tseng C.W. and Tsai C.C. (2018) Effect of proton pump inhibitors in hospitalization on mortality of patients with hepatic encephalopathy and cirrhosis but no active gastrointestinal bleeding. Clin. Res. Hepatol. Gastroenterol. 42, 353–359 10.1016/j.clinre.2017.11.011 29551615

[26] Hung T.H., Tseng C.W., Lee H.F. and Tsai C.C. (2018) Effect of Proton Pump Inhibitors on Mortality in Patients with Cirrhosis and Spontaneous Bacterial Peritonitis. Ann. Hepatol. 17, 933–939 10.5604/01.3001.0012.7193 30600287

[27] Smith E.Z., Northup P.G. and Argo C.K. (2018) Predictors of Mortality in Cirrhosis Inpatients With Clostridium difficile Infection. J. Clin. Gastroenterol. 52, 747–751 10.1097/MCG.0000000000000868 28644310

[28] Tergast T.L., Wranke A., Laser H. et al. (2018) Dose-dependent impact of proton pump inhibitors on the clinical course of spontaneous bacterial peritonitis. Liver Int. 38, 1602–1613 10.1111/liv.13862 29675988

[29] Dam G., Vilstrup H., Andersen P.K., Bossen L., Watson H. and Jepsen P. (2019) Effect of proton pump inhibitors on the risk and prognosis of infections in patients with cirrhosis and ascites. Liver Int. 39, 514–521 10.1111/liv.14012 30472808

[30] De Roza M.A., Kai L., Kam J.W. et al. (2019) Proton pump inhibitor use increases mortality and hepatic decompensation in liver cirrhosis. World J. Gastroenterol. 25, 4933–4944 10.3748/wjg.v25.i33.4933 31543684 PMC6737311

[31] Hung T.H., Tseng C.W., Tsai C.C. and Lee H.F. (2019) Effect of proton pump inhibitors on mortality of cirrhotic patients with pneumonia. PLoS ONE 14, e0216041 10.1371/journal.pone.0216041 31022265 PMC6483244

[32] Janka T., Tornai T., Borbely B. et al. (2020) Deleterious effect of proton pump inhibitors on the disease course of cirrhosis. Eur. J. Gastroenterol. Hepatol. 32, 257–264 31464790 10.1097/MEG.0000000000001499

[33] Lewis D.S., Lee T.H., Konanur M. et al. (2019) Proton Pump Inhibitor Use Is Associated with an Increased Frequency of New or Worsening Hepatic Encephalopathy after Transjugular Intrahepatic Portosystemic Shunt Creation. J. Vasc. Interv. Radiol. 30, 163–169 10.1016/j.jvir.2018.10.015 30638914

[34] Nardelli S., Gioia S., Ridola L., Farcomeni A., Merli M. and Riggio O. (2019) Proton Pump Inhibitors Are Associated With Minimal and Overt Hepatic Encephalopathy and Increased Mortality in Patients With Cirrhosis. Hepatology 70, 640–649 10.1002/hep.30304 30289992

[35] Stroup D.F., Berlin J.A., Morton S.C. et al. (2000) Meta-analysis of observational studies in epidemiology: a proposal for reporting. Meta-analysis Of Observational Studies in Epidemiology (MOOSE) group. JAMA 283, 2008–2012 10.1001/jama.283.15.2008 10789670

[36] Higgins J. and Green S. (2011) Cochrane Handbook for Systematic Reviews of Interventions Version 5.1.0. The Cochrane Collaboration www.cochranehandbook.org

[37] Wells G.A., Shea B., O'Connell D. et al. (2010) The Newcastle-Ottawa Scale (NOS) for assessing the quality of nonrandomised studies in meta-analyses. http://www.ohri.ca/programs/clinical_epidemiology/oxford.asp

[38] Higgins J.P. and Thompson S.G. (2002) Quantifying heterogeneity in a meta-analysis. Stat. Med. 21, 1539–1558 10.1002/sim.1186 12111919

[39] Patsopoulos N.A., Evangelou E. and Ioannidis J.P. (2008) Sensitivity of between-study heterogeneity in meta-analysis: proposed metrics and empirical evaluation. Int. J. Epidemiol. 37, 1148–1157 10.1093/ije/dyn065 18424475 PMC6281381

[40] Egger M., Davey Smith G., Schneider M. and Minder C. (1997) Bias in meta-analysis detected by a simple, graphical test. BMJ 315, 629–634 10.1136/bmj.315.7109.629 9310563 PMC2127453

[41] Coughlin S.S. (1990) Recall bias in epidemiologic studies. J. Clin. Epidemiol. 43, 87–91 10.1016/0895-4356(90)90060-3 2319285

[42] Fujimori S. (2015) What are the effects of proton pump inhibitors on the small intestine? World J. Gastroenterol. 21, 6817–6819 10.3748/wjg.v21.i22.6817 26078557 PMC4462721

[43] Sieczkowska A., Landowski P., Zagozdzon P., Kaminska B. and Lifschitz C. (2017) The association of proton pump inhibitor therapy and small bowel bacterial overgrowth in children. Eur. J. Gastroenterol. Hepatol. 29, 1190–1191 10.1097/MEG.0000000000000946 28800034

[44] Pijls K.E., Jonkers D.M., Elamin E.E., Masclee A.A. and Koek G.H. (2013) Intestinal epithelial barrier function in liver cirrhosis: an extensive review of the literature. Liver Int. 33, 1457–1469 23879434 10.1111/liv.12271

[45] Runyon B.A., Squier S. and Borzio M. (1994) Translocation of gut bacteria in rats with cirrhosis to mesenteric lymph nodes partially explains the pathogenesis of spontaneous bacterial peritonitis. J. Hepatol. 21, 792–796 10.1016/S0168-8278(94)80241-6 7890896

[46] Bajaj J.S., Cox I.J., Betrapally N.S. et al. (2014) Systems biology analysis of omeprazole therapy in cirrhosis demonstrates significant shifts in gut microbiota composition and function. Am. J. Physiol. Gastrointest. Liver Physiol. 307, G951–G957 10.1152/ajpgi.00268.2014 25258407 PMC4233285

[47] Gupta A., Dhiman R.K., Kumari S. et al. (2010) Role of small intestinal bacterial overgrowth and delayed gastrointestinal transit time in cirrhotic patients with minimal hepatic encephalopathy. J. Hepatol. 53, 849–855 10.1016/j.jhep.2010.05.017 20675008

[48] Hu W., Tong J., Kuang X., Chen W. and Liu Z. (2018) Influence of proton pump inhibitors on clinical outcomes in coronary heart disease patients receiving aspirin and clopidogrel: A meta-analysis. Medicine (Baltimore). 97, e9638 10.1097/MD.0000000000009638 29504996 PMC5779765

[49] Malhotra K., Katsanos A.H., Bilal M., Ishfaq M.F., Goyal N. and Tsivgoulis G. (2018) Cerebrovascular Outcomes With Proton Pump Inhibitors and Thienopyridines: A Systematic Review and Meta-Analysis. Stroke 49, 312–318 10.1161/STROKEAHA.117.019166 29339434

[50] Poly T.N., Islam M.M., Yang H.C., Wu C.C. and Li Y.J. (2019) Proton pump inhibitors and risk of hip fracture: a meta-analysis of observational studies. Osteoporos. Int. 30, 103–114 10.1007/s00198-018-4788-y 30539272

